# Is utilization of health services for HIV patients equal by socioeconomic status? Evidence from the Basque country

**DOI:** 10.1186/s12939-015-0215-6

**Published:** 2015-10-29

**Authors:** Manuel García-Goñi, Roberto Nuño-Solinís, Juan F. Orueta, Francesco Paolucci

**Affiliations:** Departamento de Economía Aplicada II, Universidad Complutense de Madrid, Campus de Somosaguas, 28223 Pozuelo de Alarcón, Madrid, Spain; Deusto Business School, University of Deusto, Bilbao, Spain; Centro de Salud de Astrabudua, Osakidetza - Basque Health Service, Erandio, Spain; University of Murdoch, Perth, Australia; University of Bologna, Bologna, Italy

**Keywords:** Inequities, Socioeconomic status, ART, HIV, Universal coverage

## Abstract

**Introduction:**

Access to ART and health services is guaranteed under universal coverage to improve life expectancy and quality of life for HIV patients. However, it remains unknown whether patients of different socioeconomic background equally use different types of health services.

**Methods:**

We use one-year (2010–2011) data on individual healthcare utilization and expenditures for the total population (N = 2262698) of the Basque Country. We observe the prevalence of HIV and use OLS regressions to estimate the impact on health utilization of demographic, socioeconomic characteristics, and health status in such patients.

**Results:**

HIV prevalence per 1000 individuals is greater the lower the socioeconomic status (0.784 for highest; 2.135 for lowest), for males (1.616) versus females (0.729), and for middle-age groups (26–45 and 46–65). Health expenditures are 11826€ greater for HIV patients than for others, but with differences by socioeconomic group derived from a different mix of services utilization (total cost of 13058€ for poorest, 14960€ for richest). Controlling for health status and demographic variables, poor HIV patients consume more on pharmaceuticals; rich in specialists and hospital care. Therefore, there is inequity in health services utilization by socioeconomic groups.

**Conclusions:**

Equity in health provision for HIV patients represents a challenge even if access to treatment is guaranteed. Lack of information in poorer individuals might lead to under-provision while richer individuals might demand over-provision. We recommend establishing accurate clinical guidelines with the appropriate mix of health provision by validated need for all socioeconomic groups; promoting educational programs so that patients demand the appropriate mix of services, and stimulating integrated care for HIV patients with multiple chronic conditions.

## Introduction

The Human Immunodeficiency Virus (HIV) is a global public health issue with more than 36 million affected. The highest prevalence is found in Sub-Saharan Africa where nearly 5 % of adults live with HIV, being 69 % of all people living with HIV in the world [1]. Although HIV mortality was huge in the 1980s, the development in the 1990s of highly active antiretroviral therapy (ART) was determinant to reduce rates of death and mother-to-child transmission. However, only in developed countries, with general access to ART, HIV behaves as a chronic condition. By 2012, more than half of the HIV world population is in developing countries with inequalities in the access to ART and with a huge rate of mortality [2].

Hence, there are two completely different realities in health policy and planning when looking at HIV. First, in developed countries with mostly universal access to ART, HIV is a chronic condition. There, the challenge in health systems is to become high-performing chronic care systems [3] and efficient given the increasing evolution of health expenditures [4]. That is feasible by looking at the way in which health services are provided [5] to chronic patients and to whether patients are suffering more than one chronic condition at the same time [6] in order to adapt their demand to their need. Here, it remains unknown whether there is equity in the use of health services by patients of different socioeconomic background even when access is guaranteed by the health system. Second, in low and middle-income countries where the challenge is to increase the access to ART [7]. Worldwide, inequity in access is a crucial challenge dealing with HIV. However, most studies look at inequity from the second perspective, focusing on increasing international rates of access to ART where it is needed [8], its role on prevention [9], on mortality [10], its impact in different groups of individuals as sex workers [11] or injecting drug users [12], or early diagnosis in infants [13]. We contribute to the literature by developing a different analysis looking at the first mentioned reality: HIV health policy and planning in developed countries where access to ART is guaranteed.

We look at the Basque Country, a region in Spain, where access to public healthcare services is universal and free at the point of use, where ART is free and patients only pay a copayment rate in other pharmacological treatments [14]. We utilize individual data from the entire Basque population (2.26 million inhabitants) on diagnoses, socioeconomic information and standardized health expenditures. We test whether patients of different socioeconomic background equally use different types of health services.

### Data and methods

We utilize the database prepared by the population stratification program (PREST) of the Basque Country including the practical totality of its population: every individual covered on 31 August 2011 by the public health insurance in the Basque Country and who was covered for at least 6 months in the previous year, regardless of whether they made any contact with or use of the Basque Health Service. The analysis refers to one year, from September 1^st^, 2010 to August 31^st^, 2011. There are 2,262,698 individuals, being 50.90 % female. As for the age distribution, 15 % are children (younger than 18) and 20 % are over 65, being the average individual 43.69 years old. It is therefore important to remark that it is not a random sample but the real population in terms of health policy and planning.

Our dataset combines three types of information. First, diagnoses information is based on hospital discharges, emergency department, primary care medical records, and prescriptions. They all are coded according to the ICD-9-CM [15] (diagnoses) and ATC [16] (pharmaceuticals). Second, out of utilization we obtain individual standardized health expenditures. Third, we utilize socioeconomic information. For the sake of our analysis, we only look at the HIV diagnosis in order to differentiate among HIV patients and non-HIV reported individuals. With respect to health expenditures, the cost of the public health services provision is based on use. However, there are no market prices within the Basque Health Service and costs are estimated through standardization of total health expenditures per type of service. We take into account the number of visits to primary care, specialist care, Accident & Emergency, rehabilitation sessions, outpatient care, laboratory tests, radiological examinations, and various outpatient procedures such as dialysis, radiotherapy and chemotherapy. Cost of hospitalization and outpatient surgery is assigned through the cost-weights of the corresponding diagnosis-related groups (DRGs). Finally, the cost of ART, provided in public hospitals in the Basque Country, has been calculated as the average cost of all ART provided in 2012. Total number of patients with ART was 5002 and total expenditure in ART treatment was of 38501376€, for an average cost per treatment of 7697€. Finally, the cost of pharmaceutical prescriptions (excluding ART) recorded in electronic health records is based on market prices. Information on socioeconomic status is derived out of the deprivation index (DI), an ordinal variable elaborated for Spain in 2008 [17] categorizing into five socioeconomic groups (SEG) by quintiles. The DI allows for the estimation of socioeconomic and environmental inequities among inhabitants by censal code. It takes into account five dimensions including the percentages of residents who are manual workers, unemployed, temporary employees, or have an inadequate level of educational attainment, overall and also specifically among young people.

We use OLS regressions to identify whether there are inequities in the use of of health services provision of any type for HIV patients in a population in which access to ART and other treatments is free and granted. Following the risk adjustment literature, our dependent variable is health expenditures for HIV patients on age groups, which, as mentioned above, is directly related to the utilization of health services (standardized health expenditures by use). With respect to our independent variables, because we do not observe all variables that might affect health expenditures we use fixed effect by socioeconomic groups (SEG). We do the same for the different types of health expenditures (provision) and for total health expenditures. In that further estimation approach, we take into account the individual age and the number of chronic conditions suffered as the risk adjustment literature states that greater need of health provision derives in greater expected health expenditure [18]. The aim is to control for healthcare needs, taking the individual number of comorbidities as a proxy. In order to construct those variables, we utilize a list of 52 health conditions defined by the research team based on the related literature [19–21]. We capture the role of multimorbidities by defining three categories: 1 to 3 comorbidities, 4 to 6, and 7 or more. The specification for our estimation model is given by:

$$ Health\kern0.5em  Expenditure{s}_i={\displaystyle \sum_j{\alpha}_j ag{e}_{ij}+{\displaystyle \sum_k{\beta}_kSE{G}_{ki}+{\displaystyle \sum_l{\delta}_l NMor{b}_{li}+{\varepsilon}_i}}} $$

## Results

Table 1 presents a description of the Basque Country population and its prevalence of HIV as a function of the demographic, socioeconomic characteristics, and by gender and age groups subject to the PREST dataset. Prevalence for the total population is of 1.164 per 1000 individuals. HIV prevalence is more than double for males than for females (1.616 vs 0.729) although for young individuals from 16 to 25, females present a greater prevalence (0.245 vs 0.192). More than half of all HIV-reported patients belong to the group of 46 to 65 years old (1361 out of 2635), being also important the group of those from 26 to 45 (1135 cases). The remaining age groups also have HIV patients although prevalence is very small. Interestingly, we observe the process of ageing in the HIV population with 8 cases of HIV patients older than 80. Finally, HIV prevalence decreases with socioeconomic status (being from richest to poorest SEG 0.784, 0.819, 1.095, 1.114, and 2.135 respectively), with significant differences between the first two SEG, the third and fourth, and the poorest SEG that presents a prevalence three times that of the richest SEG and almost double that of the total population. Socioeconomic status makes a difference in prevalence for age groups older than 26, being highest for the poorest SEG between 46 and 65 years old (prevalence ratio of 4.208).

**Table 1:** HIV prevalence ratio by socio-demographic groups in the Basque Country

	Target population		HIV		Prevalence ratio per 1000 population	Prevalence with respect to the population average
	N	%	N	%		
All	2,262,698	100	2635	100	1.165	1.00
Sex groups						
Males	1,111,050	49.10	1796	68.16	1.616	1.39
Females	1,151,648	50.90	839	31.84	0.729	0.63
Age groups						
Age 0 to 15	305,573		16		0.052	0.04
Males	157,884	51.67	10	62.50	0.063	0.05
Females	147,689	48.33	6	37.50	0.041	0.03
Age 16 to 25	192,636		42		0.218	0.19
Males	98,802	51.29	19	45.24	0.192	0.17
Females	93,834	48.71	23	54.76	0.245	0.21
Age 26 to 45	715,995		1135		1.585	1.36
Males	366,970	51.25	702	61.85	1.913	1.64
Females	349,025	48.75	433	38.15	1.241	1.07
Age 46 to 65	621,406		1361		2.190	1.88
Males	307,222	49.44	1010	74.21	3.288	2.82
Females	314,184	50.56	351	25.79	1.117	0.96
Age 66 to 80	298,687		73		0.244	0.21
Males	135,878	45.49	50	68.49	0.368	0.32
Females	162,809	54.51	23	31.51	0.141	0.12
Age over 80	128,401		8		0.062	0.05
Males	44,294	34.50	5	62.50	0.113	0.10
Females	84,107	65.50	3	37.50	0.036	0.03
Socioeconomic groups						
First SEG	479,316		376		0.784	0.67
Males	228,747	47.72	252	67.02	1.102	0.95
Females	250,569	52.28	124	32.98	0.495	0.42
Second SEG	487,140		399		0.819	0.70
Males	238,951	49.05	277	69.42	1.159	1.00
Females	248,189	50.95	122	30.58	0.492	0.42
Third SEG	457,665		501		1.095	0.94
Males	226,345	49.46	353	70.46	1.560	1.34
Females	231,320	50.54	148	29.54	0.640	0.55
Fourth SEG	422,729		471		1.114	0.96
Males	209,966	49.67	309	65.61	1.472	1.26
Females	212,763	50.33	162	34.39	0.761	0.65
Fifth SEG	415,848		888		2.135	1.83
Males	207,041	49.79	605	68.13	2.922	2.51
Females	208,807	50.21	283	31.87	1.355	1.16
All	2E + 06	100	2635	100	1.165	1.00
Socioeconomic groups						
First SEG	479,316		376		0.784	0.67
Age 0 to 15	67,569	14.10	3	0.80	0.044	0.04
Age 16 to 25	45,043	9.40	1	0.27	0.022	0.02
Age 26 to 45	137,891	28.77	149	39.63	1.081	0.93
Age 46 to 65	140,490	29.31	206	54.79	1.466	1.26
Age 66 to 80	60,085	12.54	16	4.26	0.266	0.23
Age over 80	28,238	5.89	1	0.27	0.035	0.03
Second SEG	487,140		399		0.819	0.70
Age 0 to 15	69,980	14.37	4	1.00	0.057	0.05
Age 16 to 25	42,220	8.67	10	2.51	0.237	0.20
Age 26 to 45	152,153	31.23	160	40.10	1.052	0.90
Age 46 to 65	135,980	27.91	205	51.38	1.508	1.29
Age 66 to 80	60,097	12.34	18	4.51	0.300	0.26
Age over 80	26,710	5.48	2	0.50	0.075	0.06
Third SEG	457,665		501		1.095	0.94
Age 0 to 15	65,088	14.22	3	0.60	0.046	0.04
Age 16 to 25	37,706	8.24	10	2.00	0.265	0.23
Age 26 to 45	146,271	31.96	210	41.92	1.436	1.23
Age 46 to 65	125,084	27.33	269	53.69	2.151	1.85
Age 66 to 80	58,785	12.84	9	1.80	0.153	0.13
Age over 80	24,731	5.40	0	0.00	0.000	0.00
Fourth SEG	422,729		471		1.114	0.96
Age 0 to 15	47,674	11.28	3	0.64	0.063	0.05
Age 16 to 25	34,220	8.10	11	2.34	0.321	0.28
Age 26 to 45	138,400	32.74	205	43.52	1.481	1.27
Age 46 to 65	115,524	27.33	242	51.38	2.095	1.80
Age 66 to 80	61,759	14.61	7	1.49	0.113	0.10
Age over 80	25,152	5.95	3	0.64	0.119	0.10
Fifth SEG	415,848		888		2.135	1.83
Age 0 to 15	55,262	13.29	3	0.34	0.054	0.05
Age 16 to 25	33,447	8.04	10	1.13	0.299	0.26
Age 26 to 45	141,280	33.97	411	46.28	2.909	2.50
Age 46 to 65	104,328	25.09	439	49.44	4.208	3.61
Age 66 to 80	57,961	13.94	23	2.59	0.397	0.34
Age over 80	23,570	5.67	2	0.23	0.085	0.07

Provided their health service utilization, the Basque population presents an average total health expenditures of 1132€, being of 1118€ for non-HIV (reported) individuals, and of 13260€ for HIV patients. Table 2 shows expenditures by type of health service and for non-HIV and HIV patients. Hospitalization is the most expensive type of health service (372€), being the second Primary Care (261€) and the third specialist care (255€). Differently, ART (7687€) is the most expensive type of provision for HIV patients, being second hospitalization (3372€), and third specialist care (1334€). Although for every type of provision HIV patients are more expensive than non-HIV in absolute terms, it is worth to look at relative expenditures. ART represents 58.05 % of total health expenditures for HIV patients. However, if we take for granted ART and do not take it into account, relative weight of expenditures on hospitalization are much more important for HIV patients (60.62 % vs 33.32 %) and some greater for specialist care (23.99 % vs 22.87 %) while relative expenditures on A&E (4.83 % vs 3.25 %), primary care (23.33 % vs 6.80 %) and pharmaceutical prescriptions (15.65 % vs 5.34 %) are greater for non-HIV patients.

**Table 2:** Descriptive cost per type of health service for HIV and non-HIV individuals

	All		Not-HIV patients		HIV patients		
Cost in different types of health services	Mean (std.dev.)	%	Mean (std.dev.)	%	Mean (std.dev.)	% of total expenditure with ART	% of total expenditure without ART
Primary Care (€)	261.14 (327.62)	23.05 %	261.00 (327.50)	23.33 %	378.19 (394.87)	2.85 %	6.80 %
Specialist Care (€)	257.08 (999.97)	22.69 %	255.82 (995.98)	22.87 %	1334.73 (2583.12)	10.07 %	23.99 %
Accidents and Emergency (€)	54.20 (142.78)	4.78 %	54.06 (142.01)	4.83 %	180.64 (438.16)	1.36 %	3.25 %
Hospitalizations (£)	376.29 (2294.01)	33.21 %	372.79 (2269.33)	33.32 %	3372.62 (9638.71)	25.43 %	60.62 %
Pharmaceutical Prescriptions (€)	175.21 (513.43)	15.47 %	175.07 (513.11)	15.65 %	297.22 (726.50)	2.24 %	5.34 %
ART Treatment {€)	8.96 (262.51)	0.79 %	0.00	0.00 %	7697.00 (0.00)	58.05 %	-
Total Cost (€)	1132.90 (3152.11)	100.00 %	1118.76 (3102.18)	100.00 %	13260.40 (11425.49)	100.00 %	100.00 %
N	2,262,698		2,260,063		2635		

**Fig. 1:**
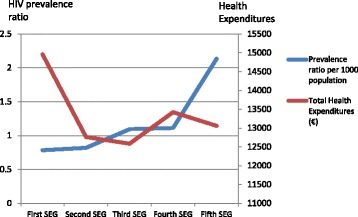
Total health expenditures and prevalence ratio by socioeconomic groups

**Fig. 2:**
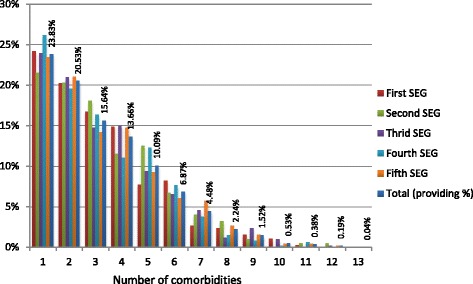
Proportion of HIV patients with different number of comorbidities by socioeconomic groups (SEG)

Table 3 presents the average cost for HIV patients by provider and SEG. The cost on ART, by construction, has been considered to be identical for all individuals. Primary care and pharmaceutical prescriptions are more used by poorer individuals while hospitalization and specialist care are more used by richest individuals. Precisely, those are the most expensive types of health provision without taking ART into account. Consequently, the Basque Country allocates a greater amount of public health resources in HIV patients in the richest SEG (14960€) while it allocates lower resources in HIV patients in the poorest SEG (13058€), and lowest for the third SEG (12585€). Figure 1 crosses the prevalence ratio and average total health expenditures for the five socioeconomic groups. While prevalence is negatively related to socioeconomic status (lower prevalence for richer SEG), total health expenditures for HIV patients is highest for the richest SEG and the trend of both series seems to be opposite. Figure 2 shows the proportion of HIV patients with different numbers of comorbidities by SEG. Even if there are some differences, the distribution of chronic conditions is similar in each SEG and for the total HIV population having about 76 % of them at least another chronic condition.

**Table 3:** Average cost per type of health expenditure for HIV patients by socioeconomic group (SEG)

	First SEG N = 376	Second SEG N = 399	Third SEG N = 501	Fourth SEG N = 471	Fifth SEG N = 888
Cost in different types of health services	Mean (std. Dev.)	Mean (std. Dev.)	Mean (std. Dev.)	Mean (std. Dev.)	Mean (std. Dev.)
Primary Care (€)	357.01 (352.78)	373.69 (357.68)	370.82 (371.14)	388.54 (407.68)	387.84 (432.31)
Specialist Care (€)	1656.69 (3134.08)	1266.92 (1458.59)	1123.16 (1620.72)	1340.54 (1795.35)	1345.16 (3383.37)
Accidents and Emergency (€)	178.23 (339.81)	179.46 (373.76)	159.11 (294.87)	185.16 (364.54)	191.94 (582.04)
Hospitalizations (€)	4791.08 (13013.95)	2995.06 (8318.83)	2960.87 (9434.31)	3490.69 (10122.34)	3111.33 (8226.76)
Pharmaceutical Prescriptions (€)	280.06 (805.00)	251.11 (539.34)	274.79 (648.04)	321.98 (779.25)	324.73 (775.82)
ART Treatment (€)	7697.00 (0.00)	7697.00 (0.00)	7697.00 (0.00)	7697.00 (0.00)	7697.00 (0.00)
Total Cost (€)	14960.07 (15519.86)	12763.24 (9337.31)	12585.76 (10870.28)	13423.91 (11495.67)	13058.00 (10412.49)

Our regression analysis is presented in Table 4. For every type of health provision we run different age groups as explanatory variables. We then add to the estimation the independent variables corresponding to the socioeconomic status and morbidity burden through the number of chronic conditions. Our main result show how socioeconomic status is significant for any type of health provision even controlling for healthcare need through the number of chronic diseases suffered by patients. Thus, patients of different SEG seem to utilize differently health providers: HIV patients in the richest SEG significantly use more specialist and hospital care while those in poorest SEG (4^th^ and 5^th^) utilize slightly more only on primary care and pharmaceutical prescriptions. Differences in the significant coefficients between richest and lowest SEG for hospital and specialist care expenditures are of 2072.9€ (12967.66€–10894.76€) and 388.11€ (2918.92€–2530.81€) while differences between lowest and richest SEG in primary care and pharmaceutical expenditures are of 21.64€ (990.29€–968.65€) and 31.06€ (962.66€–931.60€). Consequently, our results support the existence of inequities in the provision of health services for HIV patients by SEG once controlling for their health status. This finding is aligned with others in the literature, with specialist treatment being pro-rich within the British NHS when looking at several chronic conditions (others than HIV) [22]. With respect to our other control variables, age is only important when it is the only risk adjuster used, and the number of chronic conditions suffered by the patient explains health utilization of any type and total health expenditures, as usual in the risk adjustment literature [18], being most of the significant differences produced by the different use of hospital and specialist care. Hence, it is important to account for multimorbidity in order to understand the different level of utilization of health services by socioeconomic status, especially because patients with multiple conditions are the most expensive ones and therefore, those for whom health policy makers should pay more attention to their mix of health services provision.

**Table 4:** OLS estimations for different types of health expenditures

	Dependent variable. N=2365											
	Primary Care (€)		Specialist Care (€)		Accidents and Emergency (€)		Hospital Care (€)		Pharmaceutical Prescriptions (€)		Total Health Expenditures (€)	
	Coef. (std.dev.)	Coef. (std.dev.)	Coef. (std.dev.)	Coef. (std.dev.)	Coef. (std.dev.)	Coef. (std.dev.)	Coef. (std.dev.)	Coef. (std.dev.)	Coef. (std.dev.)	Coef. (std.dev.)	Coef. (std.dev.)	Coef. (std.dev.)
age 0 to 15	519,37** (98,10)	-	593,93 (645,69)	-	153,00 (109,43)	-	250,38 (2408,43)	-	48,91 (178,94)	-	9262,59** (2854,04)	-
age 16 to 25	252,11** (60,55)	−289,48** (107,60)	1123,02** (398,53)	538,55 (739,45)	214,93** (67,54)	51,23 (124,10)	865,42 (1486,51)	633,19 (2668,21)	27,57 (110,44)	−55,53 (195,86)	10180,05** (1761,55)	877,97 (3105,87)
age 26 to 45	345,29** (11,64)	−258,76** (92,33)	1283,64** (76,66)	404,06 (634,53)	209,21** (12,99)	−6,13 (106,49)	3132,94** (285,95)	1499,04 (2289,64)	204,92** (21,24)	6,77 (168,07)	12873,01** (338,86)	1644,99 (2665,20)
age 46 to 65	396,01** (10,63)	−238,70** (92,31)	1417,34** (70,01)	412,78 (634,41)	159,41** (11,86)	−80,22 (106,47)	3709,61** (261,13)	1412,49 (2289,21)	352,07** (19,40)	100,84 (168,04)	13731,43** (309,45)	1607,19 (2664,70)
age 66 to 80	576,20** (45,92)	−135,01 (101,58)	959,89** (302,29)	−402,81 (698,09)	136,23** (51,23)	−170,66 (117,16)	3280,82** (1127,54)	−883,57 (2518,96)	851,94** (83,77)	462,57** (184,90)	13502,08** (1336,16)	−1129,48 (2932,15)
age over 80	584,91** (138,74)	−115,25 (158,95)	543,45 (913,14)	−709,40 (1092,38)	19,12 (154,75)	−270,21 (183,34)	291,89 (3406,04)	−3089,37 (3941,69)	912,88** (253,06)	557,52 (289,34)	10049,26** (4036,22)	−3626,71 (4588,25)
First quintile SEG1	-	968,65** (96,52)	-	2918,92** (663,35)	-	559,58** (111,33)	-	12967,66** (2393,61)	-	931,60** (175,70)	-	26043,42** (2786,23)
Second quintile SEG2	-	979,53** (96,13)	-	2506,46** (660,64)	-	552,90** (110,88)	-	11063,24** (2383,83)	-	893,35** (174,99)	-	23692,50** (2774,85)
Third quintile SEG3	-	980,46** (96,09)	-	2331,26** (660,36)	-	528,22** (110,83)	-	10917,74** (2382,84)	-	928,36** (174,91)	-	23383,04** (2773,70)
Fourth quintile SEG4	-	1009,07** (96,29)	-	2599,75** (661,78)	-	562,92** (111,07)	-	11736,13** (2387,95)	-	996,83** (175,29)	-	24601,71** (2779,64)
Fifth quintile SEG5	-	990,29** (95,60)	-	2530,81** (656,99)	-	553,94** (110,26)	-	10894,76** (2370,69)	-	962,66** (174,02)	-	23629,46** (2759,55)
Suffering from 1 to 3 chronic conditions	-	−465,86** (25,34)	-	−1979,07** (174,20)	-	−398,60** (29,23)	-	−11237,24** (628,60)	-	−890,57** (46,14)	-	−14971,34** (731,71)
Suffering from 4 to 6 chronic conditions	-	−280,49** (26,61)	-	−1362,34** (183,47)	-	−270,03** (30,79)	-	−8513,50** (662,03)	-	−602,03** (48,59)	-	−11028,40** (770,62)
Suffering more than 7 chronic conditions	-	-	-	-	-	-	-	-	-	-	-	-
R-squared	0.4860	0.5538	0.2125	0.2548	0.1486	0.2098	0.1117	0.2133	0.1701	0.2826	0.5755	0.6373

** Significance level 2 %

## Discussion

We find evidence of inequity in the use of health services of different type in HIV patients by socioeconomic status. While rich HIV patients use in average more of hospital and specialist care, poor HIV patients use in average more of primary care and spend more in pharmaceutical products, other than the ART treatment, common to all patients. With respect to demographic variables, HIV prevalence is greater for males and at poorer socioeconomic groups. At the same time, we find evidence of the ageing and “chronification” process of the HIV population because there are patients older than 80 and most of them (76 %) suffer from other conditions.

Inequity in the access to health services and ART has been extensively studied in low-income countries and the evidence of its existence is a public concern. The access to treatment is determinant to increase quality of life and expectancy of life of HIV patients, as well as for prevention and to allow patients to be and feel part of the society and contribute to economic activity [23]. Most high-income countries, differently, present universal health systems where access to ART for HIV patients and other health services provision is guaranteed and many times, free at the point of use. That is the case in the Basque Country, a region in Spain responsible for health policy, planning, and provision for its entire population. However, we contribute to the literature finding that even when access is guaranteed, there is still inequity in the use of health services.

Hence, the fact that universal health systems ensure equal right to use health services for everyone, and that all HIV patients obtain ART, do not mean that they have achieved equity in the use of different types of health services. Our analysis does not determine which is the optimal mix of health services provision for HIV patients and it is not clear, with our data, whether there is over-provision (under-provision) for the richest (poorest) of specialist or hospital care. In fact, it cannot be directly inferred that there exists discrimination. That question is out of the scope in this paper. We add our result to the literature on the inconclusive effect of health literacy in HIV patients [24]. Our interpretation is that we might have identified the existence of some barriers that prevent poorest patients to demand equally those services even when there is universal care with free provision at the point of use. Although more research is needed to determine the existence of those barriers, we point to lack of information or a different educational level associated to each socioeconomic group that might affect the way in which they understand how to demand specialist or hospital care, or the role of the GP as a gatekeeper. A better knowledge of the marginal benefits of using health services might lead richer population to demand a greater amount of health services [25].

An important goal of universal health systems is to provide equity in the access to health services for a given level of need. That involves not only access to the same ART but also the same mix of specialist, hospital, A&E, primary care or pharmaceutical treatments. In order to reach that aim, we provide three recommendations. First, on the supply, to improve and enforce clinical guidelines for health professionals setting the type of health service provision appropriate for different levels of health need. The objective would be to reduce the discretional supply of health services and unjustifiable variability. Second, on the demand, to provide information and education programs to all HIV patients, and more specifically to those in poorer socioeconomic status about the appropriate demand of the different types of health services (when to go to primary care, specialist care, or the hospital) in order to promote the same level of use of health services. Finally, on the health system, a less fragmented organization of attention will offer a seamless transition among levels of care and improve the access of the most deprived patients to costly health services. Furthermore, we have also shown evidence on how the demand of health services by HIV patients is affected by the number of other chronic conditions they suffer and, consequently, we recommend to walk towards an integrated care model in which patients are considered as a whole, with all chronic conditions at once, instead of taking care of their multiple conditions (when that is the case) independently. This is especially important given the process of ageing and “chronification” of the HIV population. That model of integrated care might benefit from our methodology to identify especially problematic patients presenting a number of chronic conditions, which are the most expensive ones.

Unfortunately, there are some limitations in our data. First, HIV diagnosis is not complete and our data only comprehends 2635 patients. It is consequence of the historical psychological stigma of HIV patients that still makes to decrease the rate of identification of patients to about 53 % (2635 out of 5002 patients receiving ART). However, after consultation with health professionals, there is no apparent bias in the lack of diagnosis by socioeconomic or demographic groups. It is more a matter of hospital personnel providing but not registering ART for any patient. Besides, our results in prevalence are consistent with other studies related to the same population [26]. Another limitation is that we excluded from our analysis the utilization of psychiatric hospitals, home and day care, transportation, prostheses, and other equipment provided to patients at home for lack of data. We suggest further research to overcome these limitations.

## Conclusions

Inequality in the access to ART is the most important global concern for public health policy makers with respect to HIV. Under a NHS, with universal coverage and mostly free at the point of use, all HIV patients have equal right to ART and other health services provision. However, our analysis shows that they do not equally use the different types of health services. We have found evidence that HIV patients in the richest socioeconomic groups utilize more specialist and hospital care than poorer patients; and HIV patients in the poorest socioeconomic groups utilize more primary care and pharmaceutical products than richer patients. Hence, there might be some barriers preventing all patients to equally demand every type of health service. While we do not state which is the optimal mix of types of health services by health need, we understand that a universal health system should search for equal provision to patients of equal need no matter their socioeconomic status. We have pointed the different mix in the demand to barriers related to information and educational levels. Our recommendations are based on implementing accurate clinical guidelines in the mix of health services provision by need, determined by experts, so that the deviations to the supply are reduced; to provide information and educational programs for all but specifically for poorer HIV patients so that they understand better when and to which health provider they should demand health provision; and to walk towards an integrated care system in which all conditions suffered by the patient are taken into account at once. Those recommendations should help policy makers in the search for equity not only in the legal access to ART and other health services, but also in their actual demand and provision by HIV patients only based on need.

### Ethics committee approval

This database is property of the Basque Health Service and the access to it is restricted. The Clinical Research Ethics Committee of the Basque Country approved this study according to the Spanish Law 14/2007 on Biomedical Research, the Ethical Principles for Medical Research of the Declaration of Helsinki and other applicable ethical principles. All individual information has previously to this study been codified in order to ensure patient confidentiality. Written consent by the patients was specifically waived by the approving Committee.

## Abbreviations

HIV: Human Immunodeficiency Virus
ART: Antiretroviral Therapy
PREST: Population Risk Stratification Program
DRGs: Diagnosis Related Groups
ICD-9-CM: The International Classification of Diseases, Ninth Revision, Clinical Modification
DI: Deprivation Index
SEG: Socioeconomic Groups
NHS: National Health Service
A&E: Accident and Emergency
